# Case Report: Nonoperative management of traumatic dual-vessel hepatic infarction

**DOI:** 10.3389/fsurg.2026.1855023

**Published:** 2026-06-01

**Authors:** Rami Madani, David M. Richter, Yaqub Ahmedfiqi, John Henriquez, Robert G. Sawyer

**Affiliations:** Department of Surgical Sciences, Western Michigan University Homer Stryker M.D. School of Medicine, Kalamazoo, MI, United States

**Keywords:** blunt liver injury, case report, dual-vessel hepatic injury, hepatic artery injury, hepatic infarction, hepatic ischemia, hepatic vein injury, nonoperative management

## Abstract

Hepatic infarction is localized hepatocyte necrosis caused by loss of arterial or portal blood flow; dual-supply infarction, involving simultaneous occlusion of both, is exceedingly rare and typically iatrogenic. Patients commonly present with abdominal pain, nausea, and marked transaminitis, often progressing to hemorrhage or sepsis. In postpartum women, symptom overlap with hepatic disorders such as HELLP syndrome may delay diagnosis. Here, we present a woman in her thirties, three weeks post-cesarean section, who developed dual-supply infarction of the left lobe after sustaining blunt abdominal trauma. Diagnosis was confirmed radiologically, and she was managed nonoperatively with close monitoring, correction of coagulopathy, and early anticoagulation. Liver enzymes normalized and follow-up imaging showed complete resolution. This represents the first documented case of dual occlusion of both inflow vessels following blunt abdominal trauma in a postpartum patient, in addition to the first documented instance of hepatic lobe atrophy with contralateral hypertrophy following traumatic devascularization alone. This case highlights that carefully selected anticoagulation can preclude the need for surgical intervention, even in traumatic dual-supply hepatic infarction.

## 1 Introduction

Hepatic infarction, defined as parenchymal necrosis due to disruption of intrahepatic blood flow, most commonly occurs due to occlusion of a single vessel—either the hepatic artery or portal vein. However, given the liver's dual perfusion from the hepatic artery and portal vein, infarction remains infrequent, and multiple insults are typically required to produce significant necrosis (1). Rarely, both the hepatic artery and portal vein may become obstructed; interestingly such dual-vessel occlusion classically occurs iatrogenically in the context of hepatic transplantation, hepatic chemoembolization, complex hepatobiliary surgery, or systemic thrombotic disorders such as antiphospholipid syndrome and sickle cell disease (2).

Patients typically present with right upper quadrant pain, nausea, vomiting, and abrupt transaminitis; however, such non-specific features may overlap with hepatic pathologies encountered in the postpartum period—e.g., acute fatty liver of pregnancy, Budd-Chiari syndrome, and HELLP syndrome—often resulting in diagnostic uncertainty (3). Management tends to be multimodal, often involving anticoagulation, corticosteroids, transfusions, antihypertensives, and, in select cases, plasma exchange or intravenous immunoglobulin. Patients often require extensive monitoring and escalation to intensive care (4). While some clinical practice guidelines exist for the management of acute liver failure and portal vein thrombosis, there are currently no established recommendations that specifically address management of acute segmental liver infarction (5).

Notably, dual-vessel occlusion after blunt trauma is extraordinarily rare; upon extensive literature review of the English-language medical literature (Supplementary 1), we were unable to locate a single documented case of such an occurrence. Here, we present, to our knowledge, the first reported case of dual occlusion of both inflow vessels after blunt abdominal trauma in a postpartum patient. Additionally, this represents the first documented case of hepatic lobe atrophy with compensatory contralateral hypertrophy following traumatic devascularization, a phenomenon previously described only in iatrogenic contexts, such as portal vein ligation.

## 2 Case description

A previously healthy woman in her early thirties presented to the Emergency Department (ED) six hours after she was assaulted by multiple individuals. She reported being kicked and punched in multiple areas, including her head, neck, abdomen, and thorax. Following an episode of bilious emesis and progressive abdominal pain, the patient elected to receive further evaluation.

The patient's medical history was significant for an uncomplicated low-transverse cesarean section (LTCS) three weeks prior. She also had two additional LTCS, one of which was complicated by postpartum hemorrhage requiring transfusion of packed red blood cells (PRBCs). Her medical and family histories were otherwise unremarkable.

On arrival, the patient was hemodynamically stable. Initial examination revealed diffuse abdominal tenderness without guarding or rebound, accompanied by generalized abdominal ecchymosis. Laboratory investigations were notable for mild transaminitis (AST 74 U/L, ALT 70 U/L), leukocytosis (22.8 × 10^9^/L), and a hemoglobin of 98 g/L. Thromboelastogram (TEG) demonstrated an R time of 3.2 min, maximum amplitude (MA) 60.8 mm, and mild ADP platelet inhibition (ADP MA 31.4 mm). A focused assessment with sonography for trauma (FAST) exam showed intraperitoneal fluid. Contrast-enhanced CT scan of the chest, abdomen, and pelvis revealed a grade IV liver injury with devascularization of the left lateral hepatic lobe, irregularity of the adjacent left main portal vein, large-volume hemoperitoneum with suspected active extravasation from the right adnexa, fat stranding in the left omentum suggestive of omental injury, and a grade I splenic injury (Figure 1). While in the ED, the patient received three units of PRBCs, six units of pooled platelets, and one unit of fresh frozen plasma (FFP) for ongoing hemorrhage.

**Figure 1:**
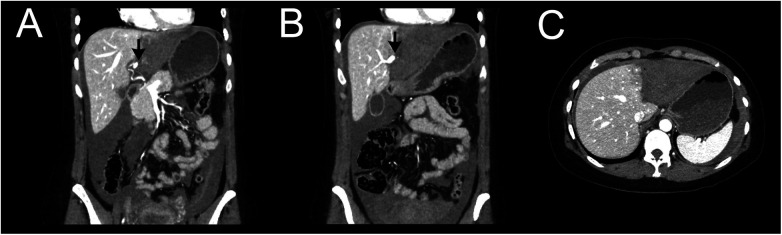
Contrast-enhanced computed tomography (CT) showing injuries on presentation. Coronal views show left main portal vein occlusion and extensive hemoperitoneum **(A)** and left hepatic artery occlusion **(B)**. Complete devascularization of the left lateral segment and part of the left medial segment can be seen on the axial view **(C)**. Arrows indicate vascular occlusion.

The patient was admitted to the Surgical Trauma Intensive Care Unit for monitoring and kept NPO. As the patient remained hemodynamically stable, a nonoperative approach was adopted; she remained under close observation and showed no signs of hemodynamic compromise or shock. However, given the patient's relative anemia, suspected arterial bleeding, and TEG abnormalities, the patient received one unit each of PRBCs, FFP, and platelets in response to relative anemia and suspected arterial bleeding. Hemoglobin responded appropriately and no further transfusions were required.

On post-injury day (PID) 2, liver function tests revealed a sharp rise in transaminases, peaking at ALT 1,026 U/L and AST 420 U/L. A three-phase contrast-enhanced CT on PID-3 confirmed infarction of the left liver lobe due to occlusion of the left hepatic artery and left portal vein; the hemoperitoneum had reduced in volume (Figure 2A). A hepatobiliary consult was obtained; in the context of no previous liver disease, it was decided that her functional liver remnant was sufficient, and no immediate surgical intervention was recommended. By PID-5, liver enzymes had largely normalized (AST 33 U/L, ALT 280 U/L), and leukocytosis had resolved; the patient also began to tolerate oral intake. At this time, given her postpartum hypercoagulable state and evidence of hepatic thrombi, anticoagulation was begun with rivaroxaban (10 mg daily), and over the remainder of her hospitalization, the patient developed progressive hypophosphatasemia—consistent with hepatocyte regeneration—but did not develop hyperbilirubinemia. The patient was discharged with rivaroxaban, along with appropriate multimodal pain management.

**Figure 2:**
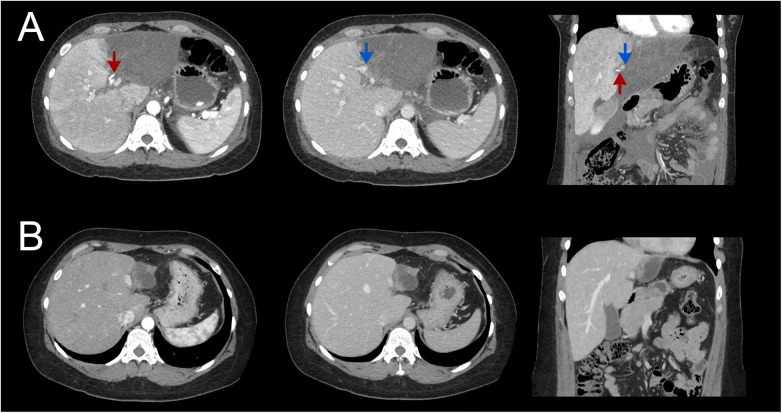
Three-phase contrast-enhanced computed tomography (CT) obtained following injury and upon follow-up. Imaging on post-injury day three **(A)** confirmed occlusion of the left hepatic artery (red) and left portal vein (blue). At nine months post-injury **(B)**, there was extensive devitalization of much of the left liver lobe, with compensatory hypertrophy of the right lobes.

The patient was scheduled for imaging and follow-up two months post-discharge but was unable to obtain the planned CT due to external social circumstances. At her two-month outpatient visit, she showed near-complete resolution of abdominal pain and had successfully returned to work without lifestyle modifications. A repeat three-phase CT was ultimately able to be performed nine months post-discharge, which showed evolving devitalization in segments II, III, and the lateral aspect of segment IV, with hypertrophy of the remaining segments (Figure 2B). Repeat laboratory evaluation showed complete normalization of the patient's hepatic panel with AST 26, ALT 25, total bilirubin 0.5, albumin 4.4, and INR of 1; Child-Pugh score was calculated to be 5. At that time, the patient was asymptomatic, had no new complaints, and was subsequently discharged from our care.

Written informed consent was obtained from the patient prior to publication of this case report. The patient conveyed satisfaction with this approach, as it allowed her to forgo the lengthier hospital stay and more intensive recovery process that is associated with major abdominal surgeries.

## 3 Discussion

Simultaneous thrombosis of both the hepatic artery and portal vein has been described only sporadically and in the context of sepsis or malignancy (5). Trauma-related dual-inflow obstruction, however, has not been previously reported and is, therefore, likely to be exceedingly rare. Such an event likely reflects a “multi-hit” scenario, wherein multiple independent factors converge to amplify thrombotic potential. In this patient, early postpartum hypercoagulability combined with vascular disruption (secondary to blunt abdominal trauma) likely precipitated thrombosis and subsequent segmental necrosis of the left liver lobe (6).

Early recognition of trauma-related hepatic infarction can be challenging. Subtle contrast attenuation defects may be mistaken for transient perfusion changes, and transaminitis is often attributed to hepatic contusion rather than evolving ischemia (7). Historically, treatment involved laparotomy for packing or vessel ligation, but such interventions carry significant risks, including rebleeding, bile leaks, and abdominal compartment syndrome (8). Accordingly, current guidance favors nonoperative management for hemodynamically stable patients (9), centered on serial contrast-enhanced CT imaging, correction of coagulopathy, and selective angioembolization (10). Within this paradigm, early anticoagulation—once hemostasis is assured—can effectively address the thrombotic load without increasing rebleeding. Low-molecular-weight heparin remains first-line, although direct oral anticoagulants (DOACs) offer favorable pharmacokinetics and lower major bleed rates for portal vein thrombosis (11).

In this case, initiation of anticoagulation was delayed due to her liver injury and ongoing hemoperitoneum. Rivaroxaban was introduced only after hemodynamic stability was confirmed and the patient had no clinical signs of continued hemorrhage (12). This cautious approach led to uneventful involution of the infarct without infections or hemorrhagic complications. Therefore, nonoperative management, including carefully timed introduction of DOACs, may be a viable strategy in select patients with high-grade hepatic injuries. However, this approach requires close multidisciplinary oversight and access to surgical intervention should instability arise (13). That said, it must be noted that the use of rivaroxaban for such purposes is not supported by a large volume of evidence and caution should be exercised in extrapolating this report to routine clinical practice. Further research is warranted to guide anticoagulation protocols for complex hepatic trauma, particularly for patients with baseline hypercoagulable states (14).

Also of note, the phenomenon of hepatic lobe atrophy and contralateral hypertrophy within the context of traumatic injury is well characterized in the setting of lobar devitalization due to surgical intervention (e.g., portal vein ligation). However, no instances of such processes appear documented in the available trauma literature. Therefore, this case also represents the first documented case of this pattern occurring following traumatic devascularization alone. The absence of such reports may reflect the rarity of successful nonoperative treatment of isolated lobar devascularization. However, the mechanism for this pattern is likely the same as what occurs following iatrogenic devascularization. Following the well-characterized “hepatostat” mechanism, the affected lobe atrophies due to increased apoptosis, while the contralateral lobe hypertrophies to compensate for the loss of functional mass, thereby maintaining the liver-to-body-weight ratio at a level sufficient for homeostasis (15). The observed contralateral hypertrophy, in conjunction with sustained normalization of hepatic synthetic function, supports this proposed mechanism in our case.

## Data Availability

The original contributions presented in the study are included in the article/Supplementary Material, further inquiries can be directed to the corresponding author/s.
