# Supplementary material for: Case Report: Nonoperative management of traumatic dual-vessel hepatic infarction
Source: Front Surg. 2026 Jun 1;13:1855023. doi: 10.3389/fsurg.2026.1855023 (PMC13265517; doi:10.3389/fsurg.2026.1855023)
Supplement: Supplementary file 1 [file Supplementaryfile1.docx]

The following search strings were employed during the literature review when preparing this manuscript.

Search string: (("Hepatic Infarction"[Mesh] OR "hepatic infarct*" OR "liver infarct*" OR "hepatic ischemia") AND (("Hepatic Artery"[Mesh] OR "hepatic artery" OR arterial) AND ("Portal Vein"[Mesh] OR "portal vein" OR portal)) AND (occlu* OR thromb* OR disrupt* OR injur* OR devasculariz*) AND ("Wounds and Injuries"[Mesh] OR trauma* OR "blunt abdominal trauma") AND ("Postpartum Period"[Mesh] OR postpartum OR puerperium OR postnatal))

Databases: PubMed

Filter(s): 1980–2026

Result(s):

- None

Search string: (("hepatic infarct*" OR "liver infarct*" OR "hepatic ischemia") AND (dual OR simultaneous OR combined OR "both vessels") AND (arterial OR "hepatic artery" OR portal OR "portal vein") AND (trauma* OR injur* OR devasculariz*) AND (postpartum OR puerperium OR pregnan*))

Databases: PubMed

Filter(s): 1980–2026

Result(s):

- El Allani L, Benlamkaddem S, Berdai MA, Harandou M. A case of massive hepatic infarction in severe preeclampsia as part of the HELLP syndrome. Pan Afr Med J. 2020 Jun 9;36:78. doi: 10.11604/pamj.2020.36.78.23302. PMID: 32774637; PMCID: PMC7386275.
